# CORVIDATA: A global dataset of morphology, ecology, sociality, and life-history in *Corvidae*

**DOI:** 10.1038/s41597-025-06471-x

**Published:** 2025-12-22

**Authors:** Claudia A. F. Wascher

**Affiliations:** Centre for Ecology, Evolution and the Environment, School of Life Sciences, Anglia Ruskin University, Cambridge, UK

**Keywords:** Animal behaviour, Zoology

## Abstract

Corvids (*Aves: Corvidae*) are renowned for their advanced cognitive abilities, complex social structures, and ecological adaptability, making them model organisms for studies in ecology, evolution, behaviour and cognition. However, comprehensive datasets integrating morphological, life-history and socio-ecological traits across the family remain scarce, limiting cross-species comparisons and evolutionary analyses. Here, I present a dataset compiling key traits for 135 corvid species worldwide. Data include morphological (e.g., body measurements, endocranial volume), life-history (e.g. age at dispersal, life span), ecological (e.g., habitat, trophic niche, geographic range) and social (e.g., sociality, breeding system) traits. This resource will facilitate comparative research on the evolutionary pressures driving corvid behaviour, support investigations into the ecological and social drivers of evolution, and advance our understanding of avian adaptation in rapidly changing environments.

## Background & Summary

Corvids (*Aves Corvidae*) are an excellent model system for research in animal behaviour, cognition, ecology, neurobiology, evolution, and urban adaptation. Corvids play critical roles in ecosystems, for example as seed dispersers and scavengers, contributing to forest regeneration, nutrient cycling, and disease control^1,2^. They show high levels of adaptability to diverse and rapidly changing environments, icluding urban habitats^3^ and thus make an ideal model system to study behavioural flexibility and resilience to environmental change. Corvids further present a valuable comparative model for understanding the neural basis of advanced cognition and vocal communication^4^.

Investigating different socio-ecological traits of a species, such as social structure^5^, breeding system^6^, habitat breath^7^, seasonal resource fluctuations^8^, and predation pressure^9^ can provide a window into evolutionary processes and adaptations to different environments, with direct implications for conservation strategies and biodiversity management. It also helps to understand drives of behavioural and cognitive adaptations, shaping problem-solving strategies and decision-making processes^10^. Despite increasing research interest in corvid behaviour and cognition^11,12^, comprehensive datasets integrating socio-ecological traits remain scarce and many studies focus on specific cognitive tasks in controlled environments^12^, often overlooking the ecological and social contexts in which these behaviours evolved^13^. Recent large-scale comparative studies have begun to clarify how specific socio-ecological factors influence behavioral traits like caching or vocal mimicry in corvids^14,15^.

Information is widely scattered in different types of sources, including hard-to-access publications^16,17^, and user-restricted web pages^18^. Thus, my main objective is to provide a comprehensive, easily accessible dataset of socio-ecological traits for the world’s *Corvidae*. This dataset is intended to provide a resource for researchers investigating the evolutionary pressures shaping corvid behaviour and cognition, facilitating cross-species comparisons and advancing our understanding of cognition.

## Methods

Scientific and common names of species were compiled from Avilist version 2025, a global checklist for birds^19^. In total, 135 species belonging to the family *Corvidae* were included in the dataset. Species-level trait data were compiled from ornithological publications and published datasets describing morphological, ecological, and social traits of corvids. Trait information was primarily derived from comprehensive datasets and major reference works, including Birds of the World^18^, AVONET: morphological, ecological and geographical data for all birds^20^, BIRDBASE: a global dataset of avian biogeography, conservation, ecology and life-history traits^21^, Global abundance estimates^22^, and the Corvids Literature Database^23^. Trait data was further extracted from targeted comparative and behavioural studies, including^14,15,24,25^. Where species were not represented in the comprehensive datasets data were supplemented from stand-alone publications identified through targeted literature searches using the Corvids Literature Database and Google Scholar. Additional sources are listed in the relevant reference columns in the dataset. All data were standardised to the species level and harmonised following the taxonomy in Avilist (2025). Continuous variables (e.g. body mass) were averaged across available records when multiple values were reported. Categorical variables (e.g. habitat, social structure) were coded based on consistent definitions across sources.

For transparency and reproducibility, primary data sources used for each species are documented within the dataset. Specific references and notes regarding data provenance are provided in the *Comments* tab and the relevant *references* column in CORVIDATA^26^.

## Data Records

The CORVIDATA dataset^26^ is archived in Zenodo, where users can access it at https://zenodo.org/records/17531251. The dataset^26^ compiles species-level information on morphological (Table 1), life-history (Table 2), socio-ecological (Table 3) and geographic (Table 4) traits across 135 corvid species. All data are provided as an Excel workbook (xlsx), including a data dictionary (metadata tab) describing each variable, its definition, source, variable type, and units, and the complete dataset (CORVIDATA tab), of morphological, ecological, social, and life-history traits of *Corvidae*. Values are either continuous numeric variables (e.g. life-history such as age at dispersal or lifespan in years; morphological data such as body mass or endocranial volume) or categorical descriptors (e.g. habitat, sociality). Missing data are indicated as ‘not reported’. Amount of missing data in different range variables ranges from 0% to 85%. Overall, 20% of data is missing and for ten trait variables more than 30% of cases were classified as not reported. Variables with high proportions of missing data were retained to preserve completeness, comparability, and the potential for future data incorporation. Despite limited coverage, these traits are biologically meaningful and align with variables reported in global avian trait databases.

**Table 1:** Summary information for the dataset of morphological traits of corvids.

Trait	Categories	N	Column name
Body measures	Beak length (culmen)	126	beak_length_culmen
	Beak length (nares)	122	beak_length_nares
	Beak width	123	beak_width
	Beak depth	123	beak_depth
	Tarsus lenght	126	tarsus_length
	Wing length	126	wing_length
	Kipp’s distance	122	kipps_distance
	Hand-wing index	122	hand_wing_index
	Tail lenght	126	tail_length
Body mass	41–1135	129	bodymass
Endocranial volume	Endocranial volume	72	endocranial_volume

Different traits and categories are shown, n refers to the number of species for which certain information is available or which fall into certain categories.

**Table 2:** Summary information for the dataset of life-history traits of corvids.

Trait	Categories	N	Column name
Age at dispersal	1–24	20	dispersal_age
	Not reported	115	
Lifespan in years	10–44	20	life_span
	Not reported	115	
Minimum incubation duration	15–21	64	min_incubation_duration
	Not reported	71	
Maximum incubation duration	16–26	48	max_incubation_duration
	Not reported	87	
Minimum days to fledging	12–42	62	min_fledging
	Not reported	73	
Maximum days to fledging	16–49	50	max_fledging
	Not reported	85	
Minimum clutch size	1–6	117	clutch_min
	Not reported	18	
Maximum clutch size	2–10	116	clutch_max
	Not reported	17	
Incubating sex	Both	6	incubation_sex
	Female	60	
	Not reported	69	
Nest type	cavity	4	nest_type
	cup, bowl	56	
	cup, bowl; cavity	1	
	cup, bowl; dome	1	
	cup, bowl; platform	10	
	dome	6	
	half cup, shallow	1	
	half cup, shallow; platform	2	
	not reported	35	
	platform	15	
	platform; half cup, shallow	2	
	sphere, globular	2	
Nest substrate	Bamboo, Pole, Tree, Shrub	4	nest_substrate
	Building, Pole, Tree, Rock, Shrub	10	
	Grass	1	
	Ground, Pole, Tree, Rock, Shrub	7	
	not reported	25	
	Pole, Building, Tree	7	
	Rock, Tree	1	
	Shrub, Bush, Vine; Tree	10	
	Tree, Rock, Building	70	

Different traits and categories are shown, n refers to the number of species for which certain information is available or which fall into certain categories.

**Table 3:** Summary information for the dataset of socio-ecological traits of corvids.

Trait	Categories	N	Column name
Scavenging	No	97	scavenging
	Not reported	6	
	Yes	32	
Sociality	Communal nests	11	sociality
	Groups	34	
	Territorial pairs	79	
	Not reported	11	
Cooperative breeding	Facultative	6	cooperative_breeding
	No	57	
	Not reported	50	
	Yes	22	
Habitat	Coastal	1	habitat
	Desert	6	
	Forest	80	
	Grassland	7	
	Human modified	7	
	Not reported	4	
	Rock	4	
	Shrubland	13	
	Woodland	13	
Trophic niche	Frugivore	4	trophic_niche
	Granivore	3	
	Invertivore	17	
	Not reported	4	
	Omnivore	107	
Primary lifestyle	Generalist	20	primary_lifestyle
	Insessorial	73	
	Not reported	4	
	Terrestrial	38	
Caching	Generalist	34	caching
	Non-cacher	3	
	Not reported	71	
	Specialist	22	
	Unclassified	5	
Vocal repertoire	2–87	104	repertoire
	Not reported	31	
Vocal mimicry	Yes	38	mimicry
	No	97	
	Not reported	7	
Ecological specialization index	0.37675071-2	134	ecological_specialization_index
	Not reported	1	

Different traits and categories are shown, n refers to the number of species for which certain information is available or which fall into certain categories.

**Table 4:** Summary information for the dataset of geographic traits of corvids.

Trait	Categories	N	Column name
Geography	Minimum latitude	122	min_latitude
	Maximum latitude	122	max_latitude
	Centroid latitude	122	centroid_latitude
	Centroid longitude	122	centroid_longitude
	Range size	122	range_size
Corvid database	0	39	corvid_database_entries
	1–422	96	
IUCN Red List Category	Critically endangered	3	2024_IUCN_Red_List_Category
	Endangered	3	
	Extinct in the wild	1	
	Least concern	100	
	Near threatened	9	
	Not reported	11	
	Vulnerable	8	
Biogeographic realm	Afrotropical	6	biogeographic_realm
	Afrotropical, Madagascar & islands	1	
	Afrotropical, Palearctic	3	
	Australian	9	
	Australian, Wallacea	1	
	Cosmopolitan	1	
	Eastern Hemisphere	1	
	Indomalayan	18	
	Indomalayan, Palearctic	11	
	Indomalayan, Palearctic, Wallacea	1	
	Indomalayan, Wallacea	3	
	Nearctic	14	
	Neotropical	34	
	Neotropical, Neacrtic	5	
	Oceania	3	
	Palearctic	17	
	Palearctic, Afrotropical	1	
	Palearctic, Indomalayan	1	
	Wallacea	5	
Latitudinal range	Temperate	36	latitudinal_range
	Tropical	63	
	Tropical-Temperate	1	
	Tropical-Temperate	35	
Elevational range	0–6000	128	elevational_range
	Not reported	7	
Abundance estimate	0-355015538	119	abundance_estimate
	Not reported	16	

Different traits and categories are shown, n refers to the number of species for which certain information is available or which fall into certain categories.

Users should note that most trait data in CORVIDATA are derived from a limited number of individuals or populations per species. Datasets like this are generally vulnerable to geograogical biases^15^. For widely distributed species, available data are often biased towards certain geographic regions, and will likely not capture the full range of intraspecific variation in morphology, life-history, behaviour, or ecology. This limitation is common to global trait databases, which rely on species-level averages to facilitate cross-species comparisons^20,21^. Researchers should interpret trait values with caution, especially for species with broad distributions or known geographic variation, and consider the potential influence of regional sampling biases in their analyses.

## Technical Validation

All species names were standardized according to the Avilist (2025) global bird checklist. Data for each species were entered manually into the dataset, and all trait values were cross-checked to ensure accuracy and reliability. Each value was extracted from a primary, peer-reviewed source. Continuous variables (e.g., morphological and life-history traits) were screened for outliers and biologically implausible values, and units were standardized (e.g., mm, g, ml, days, years). To further ensure data quality, random spot-checks were conducted across species and traits to verify data consistency against the original sources.

## Data Availability

The CORVIDATA dataset is available at https://zenodo.org/records/17531251.

## Code availability

No code has been used to generate, test, or process the current dataset.
